# Mathematical Characterization of Protein Sequences Using Patterns as Chemical Group Combinations of Amino Acids

**DOI:** 10.1371/journal.pone.0167651

**Published:** 2016-12-08

**Authors:** Jayanta Kumar Das, Provas Das, Korak Kumar Ray, Pabitra Pal Choudhury, Siddhartha Sankar Jana

**Affiliations:** 1 Applied Statistics Unit, Indian Statistical Institute, 203 B.T Road, Kolkata-700108, West Bengal, India; 2 Department of Biological Chemistry, Indian Association for the Cultivation of Science, 2A & 2B Raja S. C. Mullick Road, Kolkata-700032, West Bengal, India; 3 Department of Chemistry, Indian Institute of Technology-Bombay, IIT Bombay, Powai, Mumbai-400076, Maharashtra, India; Russian Academy of Medical Sciences, RUSSIAN FEDERATION

## Abstract

Comparison of amino acid sequence similarity is the fundamental concept behind the protein phylogenetic tree formation. By virtue of this method, we can explain the evolutionary relationships, but further explanations are not possible unless sequences are studied through the chemical nature of individual amino acids. Here we develop a new methodology to characterize the protein sequences on the basis of the chemical nature of the amino acids. We design various algorithms for studying the variation of chemical group transitions and various chemical group combinations as patterns in the protein sequences. The amino acid sequence of conventional myosin II head domain of 14 family members are taken to illustrate this new approach. We find two blocks of maximum length 6 aa as ‘FPKATD’ and ‘Y/FTNEKL’ without repeating the same chemical nature and one block of maximum length 20 aa with the repetition of chemical nature which are common among all 14 members. We also check commonality with another motor protein sub-family kinesin, KIF1A. Based on our analysis we find a common block of length 8 aa both in myosin II and KIF1A. This motif is located in the neck linker region which could be responsible for the generation of mechanical force, enabling us to find the unique blocks which remain chemically conserved across the family. We also validate our methodology with different protein families such as MYOI, Myosin light chain kinase (MLCK) and Rho-associated protein kinase (ROCK), Na^+^/K^+^-ATPase and Ca^2+^-ATPase. Altogether, our studies provide a new methodology for investigating the conserved amino acids’ pattern in different proteins.

## Introduction

All living organisms are made up of proteins. The biochemical information that resides in the protein primary sequence maintain their structure, function, and even its own stability. This biochemical information is instructed/governed by the amino acid sequences. The versatility of amino acid sequences provide the different outcomes [1]. In fact, the chemical properties of amino acids which are embedded in the primary protein sequences take the key role to determine the biological activity of the protein. In-silico techniques to analyze this information in terms of their chemical nature or structure are yet to be explored completely. In literature, the phylogenetic analyses are done from various angles and different perspectives such as multiple alignments for the selection of conserved block [2], Randomized Accelerated Maximum Likelihood (RAXML) [3], conditional Lempel-Ziv (LZ) complexity [4], tree alignment graph [5] etc. A phylogeny or evolutionary tree represents the evolutionary relationships among a group of protein sequences. The longer the branch in the horizontal dimensions of the tree, the larger is the amount of change. Trees are useful in bioinformatics, system biology and various phylogenetic comparative methods [3, 6–10]. Despite its great implication, phylogenetic tree can find only similarity analysis of amino acid sequences but not the chemical nature of amino acids in a protein. Also multiple sequence alignment of several hundred sequences always produces a log jam in respect of time and biasness [11]. Several groups have worked with reduced amino acid alphabets to tackle to the above stated problems by reducing the sample size which can perform at the same level as the full alphabets in correct pair wise alignment of sequences with regards to structure similarity but low sequence identity [12, 13]. Recently XIE et al. [14] proposed a new method by using hydropathy group of amino acids to analyze the similarity/dissimilarity of protein sequence based on the conditional probability of the protein sequence [14]. Studies are also done on using the substitution matrices from several protein blocks of aligned sequence segments resulting the characterization of related proteins [15]. However, studying only the amino acid sequences of proteins limits our scope of understanding of the similarities and differences among proteins with regard to their biochemical nature. Further analyses of the sequences considering chemical nature are required and it can be done if the sequences can be studied through the chemical nature of individual amino acid.

In this report, we propose a mathematical model where the twenty amino acids are segregated into eight groups on the basis of their chemical nature. Further, we study the transition among the eight groups of distinct chemical properties of the amino acids of conventional myosin’s head domains. Their transitions in the sequences are calculated to demonstrate the unique chemical transition pattern of amino acid clustering in each sequence of conventional myosin II family. Various patterns with repetition and without repetition of amino acids’ chemical group are enumerated. We also expand our study to another inter sub-family of Kinesin, KIF1A and find a unique common block in the neck linker region. This evolutionary conserved block is hypothesized to reveal the functional role in the conversion of chemical energy to mechanical energy. Further, our studies are expanded to different protein families and results demonstrate various common blocks in the family members have been highlighted.

## Materials and Methods

### Amino acid categorization

Twenty amino acids are categorized into eight chemical groups according to their side chain shown in Table 1.

**Table 1:** Amino acid categorization based on their chemical nature.

Chemical Nature of the Group	Amino Acids
Acidic	Aspartate (D), Glutamate (E)
Basic	Arginine (R), Histidine (H), Lysine (K)
Aromatic side chain	Tyrosine (Y), Phenylalanine (F), Tryptophan (W)
Aliphatic side chain	Isoleucine (I), Leucine (L), Valine (V), Alanine (A), Glycine (G)
Cyclic	Proline (P)
Sulfur containing	Methionine (M), Cysteine (C)
Hydroxyl containing	Serine (S), Threonine (T)
Acidic amide	Glutamine (Q), Asparagine (N)

In order to characterize the amino acid sequence, we transform these groups into numerical sequence (mapping 20 distinct amino acids into eight groups as integer 1-8 only), each amino acid from respective chemical groups are replaced by corresponding group numbers shown in Table 2. These eight chemical groups of amino acids are used to characterize the chemical nature of the sequences in various ways.

**Table 2:** Mapping Twenty standard amino acids to eight chemical groups of amino acids.

Amino Acids	D	E	R	H	K	Y	F	W	I	L	V	A	G	P	S	T	M	C	Q	N
**Numerical Value**	1		2			3			4					5	6		7		8	
**Group Name**	G1		G2			G3			G4					G5	G6		G7		G8	

### Myosin super family

**Fig 1:**
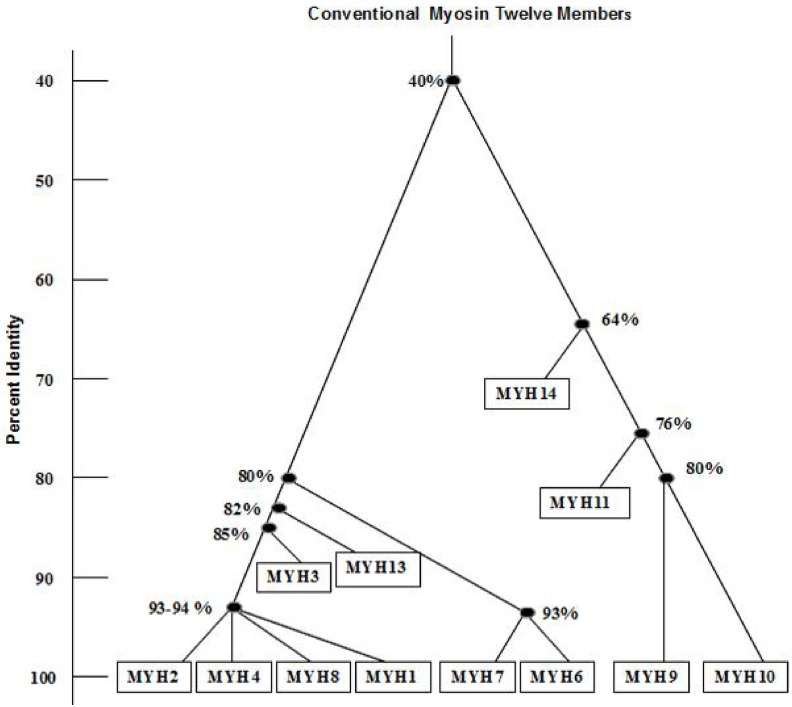
Conventional Myosin II Family as previously described in [16–18]. Based on the heavy chain amino acid sequence similarities myosin II is broadly divided into two groups; (a) non-muscle myosin group and (b) muscle myosin group as detailed in Table 3.

To analyze the effectiveness of our mathematical model we select human actin based conventional motor protein myosin II family [16–18] whose phylogenetic tree is shown in Fig 1.

Myosin II, the conventional two headed myosin protein, is composed of one pair each of heavy chain (220kDa), essential light chain (ELC, 17kDa) and regulatory light chain (RLC, 20kDa). Each heavy chain has a globular head domain containing the Actin and ATP binding domain, required for their motor activity, an intermediate domain that forms an *α*-helical coiled coil and a C-terminal non-helical tailpiece [19]. We extend the list of the human NMIIs, by adding the sequences annotated as human NMIIs sequences in uniport database. All together we collect a total of 15 candidate sequences. Most of these proteins have been assigned to a particular group by previous studies. From the new sequences, we discard MYH16 gene, as a pseudo gene [20–22]. Table 3 lists with their names, length of head domain, accession number, protein names and groups (Data is collected from www.uniport.org).

**Table 3:** Details of conventional myosin II family members in human.

Seq. Nos.	Gene Name	Length of the Head Domain (aa)	Accession Number	Protein Names and Remarks
1	MYH14	860	Q7Z406	Myosin-14(NMHC II-C)
2	MYH 11	843	P35749	Myosin-11 (SM MyHc)
3	MYH9	836	P35579	Myosin-9 (NMHC II-A)
4	MYH10	843	P35580	Myosin-10 (NMHC II-B)
5	MYH15	850	Q9Y2K3	Myosion-15
6	MYH7B	845	A7E2Y1	Myosion-7B (Cardiac *β* MyHC)
7	MYH2	844	Q9UKX2	Myosin-2 (IIa MYH2)
8	MYH4	842	Q9Y623	Myosin-4 (IIb MYH4)
9	MYH8	841	P13535	Myosin-8 (Perinatal MyHC)
10	MYH1	842	P12882	Myosin-1 (Ix/d MYH1)
11	MYH3	829	P11055	Myosin-3 (Embryonic MyHC)
12	MYH13	842	Q9UKX3	Myosin-13 (Extraocular MyHC)
13	MYH7	838	P12883	Mysion-7 (Cardiac *β* MyHC)
14	MYH6	840	P13533	Mysion-6 (Cardiac *α* MyHC)

This myosin family is having two sub groups: a) Seq.nos. 1-4 is the non-muscle myosin group, and b) Seq nos. 5-14 is the muscle myosin group. (aa-amino acid)

We chose the motor domain because these core sequences are very much conserved in all myosin classes. They consist of 7-stranded *β*-sheet encircled by several *α*-helixes. Myosin motor domain has an Actin binding region which can show open and close conformations in response to ATP binding and has a neck linker region that contains IQ motifs (IQxxxRGxxxR) responsible for myosin light chain binding [23–28].

### Myosin I family

To validate our methods with other myosin family, we chose to study MYOI class of unconventional myosin (the second biggest sub-family of myosin). Myosin Is are single headed myosins that lack the heptapeptide motif responsible for coiled-coil association in the C-terminal like myosin II [29]. There are 8 myosin I isoforms found in vertebrates, and generally referred as MYO1A-H using nomenclature adopted from Human Genome organization [30, 31]. In Table 4 shows their names, length, accession number, protein names and remarks.

**Table 4:** Details of unconventional myosin sub-family MYOI class members in human.

Seq. Nos.	Gene Name	Length of the Head Domain (aa)	Accession Number	Protein Names and Remarks
1	MYO1A	694	Q9UBC5	Unconventional myosin-Ia
2	MYO1B	701	O43795	Unconventional myosin-Ib
3	MYO1D	695	O94832	Unconventional myosin-Id
4	MYO1G	707	B0I1T2	Unconventional myosin-Ig
5	MYO1C	731	O00159	Unconventional myosin-Ic
6	MYO1H	690	Q8N1T3	Unconventional myosin-Ih
7	MYO1E	692	Q12965	Unconventional myosin-Ie
8	MYO1F	690	O00160	Unconventional myosin-If

### KIF1A class of kinesin family

To extend and validate our methods from intra-protein group to inter-protein group, we chose to study KIF1A class of kinesin protein family. Both the KIF1A class of kinesin and myosin II motor domain sequences are evolved from same ancestor [32]. Table 5 lists their names, length, accession number, protein names and remarks.

**Table 5:** Details of kinesin sub-family KIF 1A class members in human.

Seq. Nos.	Gene Name	Length of the Head Domain (aa)	Accession Number	Protein Names and Remarks
1	KIF 1A	365	Q12756	Kinesin-like protein KIF1A
2	KIF 1B	364	O60333	Kinesin-like protein KIF1B
3	KIF 1C	358	O43896	Kinesin-like protein KIF1C

### Other protein family

Further, study is also directed to by choosing different protein families such as Myosin light chin kinase (MYLK/MLCK) in Table 6 [33–35], Rho-associated protein kinase (ROCK) in Table 7 [36, 37], Na^+^/K^+^-ATPase or sodium-potassium pump enzyme in Table 8 [38, 39] and Ca^2+^-ATPase transport protein in Table 9 [40–42]. The enzymes MLCK and Rho are known for phosphorylation of the regulatory light chain of myosin II.

**Table 6:** Details of MLCK protein family members in human.

Seq. Nos.	Gene Name	Length (aa)	Accession Number	Protein Names and Remarks
1	MYLK1	1914	Q15746	Myosin light chain kinase 1
2	MYLK2	596	Q9H1R3	Myosin light chain kinase 2
3	MYLK3	819	Q32MK0	Myosin light chain kinase 3
4	MYLK4	388	Q86YV6	Myosin light chain kinase 4

**Table 7:** Details of Rho-associated protein kinase family members in human.

Seq. Nos.	Gene Name	Length (aa)	Accession Number	Protein Names and Remarks
1	ROCK1	1354	Q13464	Rho-associated protein kinase 1
2	ROCK1	1388	O75116	Rho-associated protein kinase 2

**Table 8:** Details of Na^+^/K^+^-ATPase family members in human.

Seq. Nos.	Gene Name	Length	Accession Number	Protein Names and Remarks
1	ATP1A1	1023	P05023	Sodium/potassium-transporting ATPase subunit alpha-1
2	ATP1A2	1020	P50993	Sodium/potassium-transporting ATPase subunit alpha-2
3	ATP1A3	1013	P13637	Sodium/potassium-transporting ATPase subunit alpha-3
4	ATP1A4	1029	Q13733	Sodium/potassium-transporting ATPase subunit alpha-4
5	ATP1B1	303	P05026	Sodium/potassium-transporting ATPase subunit beta-1
6	ATP1B2	290	P14415	Sodium/potassium-transporting ATPase subunit beta-2
7	ATP1B3	279	P54709	Sodium/potassium-transporting ATPase subunit beta-3
8	ATP1B4	357	B7ZKV8	Sodium/potassium-transporting ATPase subunit beta-4

**Table 9:** Details of Ca^2+^-ATPase family members in human.

Seq. Nos.	Gene Name	Length (aa)	Accession Number	Protein Names and Remarks
1	ATP2A1	1001	O14983	Sarcoplasmic/endoplasmic reticulum calcium ATPase 1
2	ATP2A2	1042	P16615	Sarcoplasmic/endoplasmic reticulum calcium ATPase 2
3	ATP2A3	1043	Q93084	Sarcoplasmic/endoplasmic reticulum calcium ATPase 3
4	ATP2B1	1258	P20020	Plasma membrane calcium-transporting ATPase 1
5	ATP2B2	1243	Q01814	Plasma membrane calcium-transporting ATPase 2
6	ATP2B3	1220	Q16720	Plasma membrane calcium-transporting ATPase 3
7	ATP2B4	1241	P23634	Plasma membrane calcium-transporting ATPase 4

### Computational algorithms

We use different algorithms for studying chemical group transitions count, pattern analysis with repetition and without repetition of chemical groups, conserved block findings etc. For all the cases separate programs are designed on using Matlab software 2013a. All the algorithms are enumerated below:

#### Algorithm 1: Chemical group transitions and ordered pair analysis

This algorithm is developed to list the total number of amino acids of certian groups (say G1) followed by the listing of the number of amino acid ordered pairs having first amino acid fixed (belonging to G1 group) and the second amino acid from every other possible groups for a given sequence.

**Input:** Set of primary protein sequences.

**Output:** Number count of chemical group transitions and lists the total number of amino acids from each group.

Firstly, amino acid sequence is transformed into numerical value (called numerical sequence having 1-8 numerals using Table 2). Initially a null matrix (G) of size 8 by 8 is generated for each sequence. Pair wise (say (*x*, *y*)) numerical sequence is read from left to right till the end of the sequence and the corresponding (*x*, *y*) cell value (*i*th row and *j*th column) of the matrix (G) is incremented by one. The cell (*i*, *j*) value of the final G matrix represents the number count from *i* to *j* chemical group transitions (or number count of (*i*, *j*) or (*Gi*, *Gj*) ordered pairs) for a particular sequence. On the other hand, every row value (a particular group) of the matrix tells about the number of transitions at which one amino acid (of one group) in a sequence changes as ordered pair to other possible groups including the self group. Every row value count (aggregate) from the matrix G is stored in column wise into another matrix (F) listing the total number of amino acids of certian groups followed by the listing of the number of amino acid ordered pairs having first amino acid fixed and the second amino acid from every other possible groups for a given sequence providing a hyphen (-) between them. Finally, we get the F matrix of size *m* × *n*; where *n* is the total number of sequences and *m* is the number of various chemical groups (for our analysis m = 8).

#### Algorithm 2: Common pattern finding without repetition of chemical groups

This algorithm is developed to find the various lengths of chemical patterns without repetition of chemical groups and their location in the sequence.

**Input:** Set of primary protein sequences and pattern length (L).

**Output:** Common pattern with amino acid sequence and location.

Firstly, as in Algorithm 1 amino acid sequence is transformed into numerical sequence (using Table 2). Given an input of pattern length L (2 ≤ *L* ≤ 8), all possible combination of pattern of length L using the numerical numbers 1-8 are generated without repetition of same numerals. Every possible pattern is investigated among the sequences, and if the pattern is found for all the sequences, the pattern is selected and stored along with the locations, otherwise the pattern is discarded. By varying pattern length L, we can search for different lengths of patterns discussed above. Finally, based on the selected patterns and their locations, corresponding primary sequence is obtained.

#### Algorithm 3: Common pattern finding with the repetition of chemical groups

This algorithm is developed to find the various lengths of chemical patterns like Algorithm 2 but with the repetition of chemical groups and their location in the sequence. To reduce the time complexity for generating the pattern of bigger length, here the concatenation technique is used starting with 4 length patterns.

**Input:** Set of primary protein sequences, common motif /pattern (*L* ≥ 4).

**Output:** Common motif/pattern with amino acid sequence and location.

Firstly, amino acid sequence set is transformed into numerical sequence like Algorithm 1. The required length of the common motifs (L) is then divided by 4 and the quotient (q) and remainder (r) are noted. All possible tetrameric permutations of 1-8 (with repetitions) (e.g. 1111, 1112… 8887, 8888) are generated, and searched in the transformed sequences. The tetramers present in all the sequences are stored, and the rest are discarded. In the next iteration, all possible tetramers of 1-8 (generated at the start) are concatenated to the stored tetrameric sequences and the resulting motif is found out.

#### Algorithm 4: Similarity analysis of pattern/block

This algorithm is developed to find out the highest percentage of similarity (with regards to chemical group) of a pattern/block from the given set of sequences and the corresponding location in the sequence.

**Input:** Set of primary protein sequences, inputted pattern/block sequence.

**Output:** Highest percentage similarity of the block with the given set of primary sequences along with multiple occurrences on each of the patterns and their locations.

Firstly, the inputted pattern/ block of length L and the set of amino acid sequences are taken out and transformed into the numerical sequences (using Table 2). L length pattern is considered as window of size L. Every numerical sequence is read from left to right equal to the inputted block of window size L and records the pairwise number of matching count m (say). Window is sliding to the next L length block right shifted by one; update the current record count (m) if the similarity matching is found more than the previous value. This is done for the last block of window. The records for the highest similarity matches and their positions in the sequences are noted. Finally, the percentage of similarity matching = (*m*/*L*) × 100) is calculated. Based on the inputted block and their locations, primary protein sequence of the block is obtained.

**Illustration:** Two amino acids sequence blocks “DRSMYI” and “EKTCWV” are transformed into the same numerical sequence “127634” as D/E, R/K, S/T, M/C, Y/W, I/V coming from same chemical group though they are different amino acids. Similarity matching of these two blocks is done based on alignment of two blocks. For an example, the above two blocks “DRSMYI” and “EKTCWV” are 100% similar (6 positions) with regards to chemical groups as position wise they are from same chemical group. Now if the first amino acid ‘D’ from the first block is replaced by Q i.e. “QRSMYI” then two blocks are approximately 84% similar ((6-1/6)*100) or by the alignment of these blocks 5 positions amino acids are from same chemical group and another one (first amino acid) is from different chemical groups.

## Results

### Percent identity matrix of a NMII family

**Fig 2:**
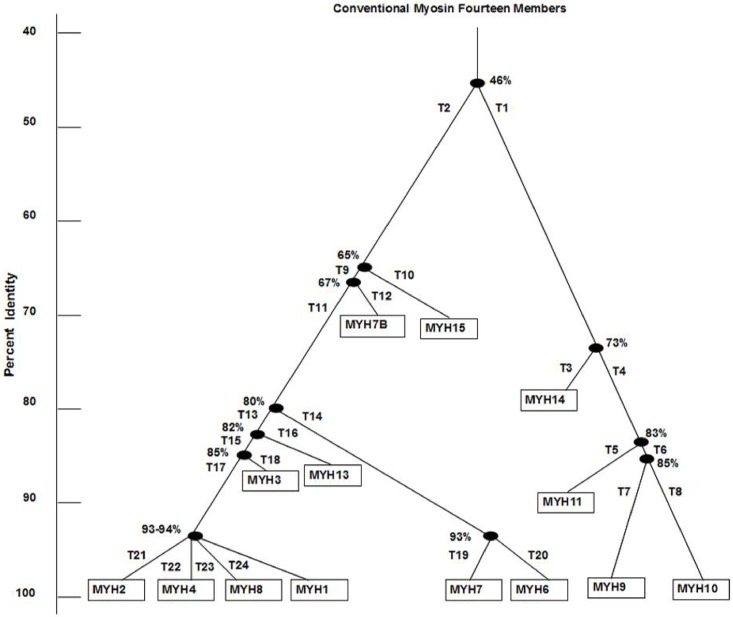
A rooted phylogenetic tree based on percent sequence similarity analysis of myosin heavy chain II head domain of humans.

We use www.uniport.org database to find myosin II sequences. MYH15 and MYH7B are not listed in the previous phylogenetic tree. To find a correct position of two newly sequenced myosin II (MYH15 and MYH7B) in the existing phylogenetic tree, we deploy pair wise percent identity matrix shown in Table 10 of the myosin head domain (839-860 aa) for every pair of sequences of the myosin II fourteen members (sequence number in serial from Table 3). The rooted phylogenetic tree (Fig 2) of the myosin fourteen members is obtained from http://www.ebi.ac.uk/Tools/msa/clustalw2/.

**Table 10:** Percentage identity matrix of every pair sequences of myosin II head domain by using the site www.ebi.ac.uk/Tools/msa/clustalw2/.

**Seq. Vs. Seq.**	**1**	**2**	**3**	**4**	**5**	**6**	**7**	**8**	**9**	**10**	**11**	**12**	**13**	**14**
1	100.0	73.21	76.47	78.57	45.47	49.04	47.29	46.56	47.83	47.17	48.61	48.25	47.34	46.02
2	73.21	100.0	84.33	83.51	46.86	49.88	49.57	49.57	50.37	49.82	50.31	50.06	49.88	48.66
3	76.47	84.33	100.0	85.77	48.01	49.08	48.77	48.77	49.20	48.89	50.37	50.12	48.95	48.21
4	78.57	83.51	85.77	100.0	47.72	49.39	49.08	48.35	49.63	48.71	50.55	49.57	50.12	47.80
5	45.47	46.86	48.01	47.72	100.0	68.95	63.15	64.14	63.34	62.70	66.18	66.27	65.06	64.30
6	49.04	49.88	49.08	49.39	68.95	100.0	65.76	65.91	66.99	65.79	69.26	68.38	66.75	65.55
7	47.29	49.57	48.77	49.08	63.15	65.76	100.0	92.28	93.92	93.59	79.47	79.52	85.94	85.75
8	46.56	49.57	48.77	48.35	64.14	65.91	92.28	100.0	92.36	95.49	79.95	80.45	85.08	87.02
9	47.83	50.37	49.20	49.63	63.34	66.99	93.92	92.36	100.0	93.32	80.62	80.55	86.17	86.16
10	47.17	49.82	48.89	48.71	62.70	65.79	93.59	95.49	93.32	100.0	80.31	80.21	84.84	85.95
11	48.61	50.31	50.37	50.55	66.18	69.26	79.47	79.95	80.62	80.31	100.0	90.69	79.07	79.19
12	48.25	50.06	50.12	49.57	66.27	68.38	79.52	80.45	80.55	80.21	90.69	100.0	79.24	79.83
13	47.34	49.88	48.95	50.12	65.06	66.75	85.94	85.08	86.17	84.84	79.07	79.24	100.0	82.46
14	46.02	48.66	48.21	47.80	64.30	65.55	85.75	87.02	86.16	85.95	79.19	79.83	82.46	100.0

Based on the head domain amino acid sequence, MYH15 has almost 65% identity with other nine members of the left sub-tree and MYH7B has almost 67% identity with other eight members of the left sub-tree. Positions of these two members in the phylogenetic tree are shown in Fig 2. At the branching point, left sub-tree and right sub-tree symbolized with T1, T2…T24 where T1 implies sequence no. 1-4 and T2 implies sequence no. 6-14 from Table 3 and so on. Our current analyses from the percent identity matrix (Table 10) for the fourteen members of the myosin head domain suggest that the first branching point (T) is at 46%, almost close to the previous reports [16, 17], which is 40%. Furthermore deviations are found for the right branch of the phylogenetic tree at the breaking point at T3 (73% vs. 64%), at T5 (83% vs. 76%) and at T7 (85% vs. 80%). It is interesting to note that there is not much deviation for any breaking point found for the left branch of the phylogenetic tree i.e. among the sequences 5-14.

### Chemical group transitions for the fourteen members of myosin II family head domain

In line with the conventional term “Substitution Matrix”used in Bioinformatics and evolutionary Biology, our Algorithm 1 describes the number of transitions at which one amino acid (Say G1) in a sequence changes to other possible groups (G1,G2,…,G8) including the self-group as ordered pairs. This is done while looking for the similarity of the protein sequences. We hereby describe the distinct chemical group transitions for every protein sequences of myosin II family. Group transition is the transition of one chemical group to other possible chemical groups; like from G1 (acidic) to G2 or others. Theses transitions or ordered pairs are counted and recorded in numbers (S1 Table) for every sequence and aggregate transition for one particular group to all other groups are shown in Table 11.

**Table 11:** List the total number of amino acids from a particular chemical group followed by the order pairs count except self order pair for each sequence of myosin head domain.

Seq. Nos.	#G1-#X1	#G2-#X2	#G3-#X3	#G4-#X4	#G5-#X5	#G6-#X6	#G7-#X7	#G8-#X8
1	102-90	129-114	76-71	311-192	48-43	31-31	77-70	85-81
2	106-89	129-114	81-75	290-191	27-24	38-37	83-74	88-83
3	106-91	132-118	81-75	290-184	30-27	38-36	67-64	91-85
4	107-93	138-121	84-78	285-191	29-26	38-37	72-68	89-84
5	103-88	127-108	92-83	298-199	25-23	42-41	82-75	80-74
6	99-88	129-113	88-82	292-186	34-31	37-34	81-78	84-78
7	102-89	130-114	89-83	290-192	29-27	33-31	93-83	77-72
8	102-89	129-112	91-85	281-189	31-29	38-35	93-85	76-69
9	100-87	129-113	91-85	280-192	30-28	36-34	98-86	76-70
10	101-88	128-112	90-84	283-190	30-28	38-35	93-83	78-70
11	103-89	134-116	90-85	274-187	29-27	35-34	98-87	75-68
12	102-90	126-109	91-85	272-180	31-29	45-41	89-78	85-76
13	102-88	131-116	89-83	285-187	32-30	36-35	82-74	80-72
14	103-88	131-115	89-82	284-190	27-25	37-36	83-76	85-77

Here, #Gi is the number count of amino acids from Gi chemical group and #Xi is the the number count of order pairs except the pair (Gi,Gi).

### Phylogenetic tree analysis for the fourteen members of Myosin II head domain

It is observed that based on one or more chemical group transitions with their distinct ranges in numbers, every branches of the phylogenetic tree can be explained clearly as shown in Table 12. For illustration, at branching point 46% identity (Fig 2) where left sub-tree T2 having sequences 5-14 and right sub-tree (T1) having sequences 1-4. For example, if we look on the Table 11 in column no. 4, G3 group to other group transitions number ranges for T2 are a) 88-92 with self-transition and b) 82-85 without self-transition, and ranges for T1 are a) 76-84 with self-transition and b) 71-78 without self-transition. Therefore, we found the distinct ranges value of T2 branch which is higher than T1 branch i.e. muscle group are more aromatic than the non-muscle group. One can study the significance of amino acids for this aromatic side group of myosin II family.

**Table 12:** Distinct ranges of every branch of the phylogenetic tree obtained from Table 11, percentage (%) identity of each branching point from Fig 2.

Percent identity of original sequence (%)		Hitting Groups	Distinct Range in Respective Sub-Tree				Sequences Comparison
From Fig 1	From Fig 2		Left sub-tree		Right sub-tree		
40%	46%	**G3**	T2	a) 88-92 b) 82-85	T1	a) 76-84 b) 71-78	(1-4) Vs. (5-14)
64%	73%	G3, G4, **G5**, G6	T3	a) 48 b) 43	T4	a) 27-30 b) 24-26	(1) Vs. (2-4)
76%	83%	G2, G5, **G7**, G8	T5	a) 83 b) 74	T6	a) 67-72 b) 64-68	(2) Vs. (3-4)
80%	85%	All, **G4**	T7	a) 290 b) 184	T8	a) 285 b) 191	(3) Vs. (4)
-	65%	G2, G3, G4, **G5**	T9	a) 25 b) 23	T10	a) 27-34 b) 25-31	(5) Vs. (6-14)
-	67%	G1, **G5**	T11	a) 34 b) 31	T10	a) 27-29 b) 25-29	(6) Vs. (7-14)
80%	80%	**G4**	T13	a) 89-93 b) 78-87	T14	a) 82-83 b) 74-76	(7-12) Vs. (13-14)
82%	82%	**G2**, G4, G6, G7, G8	T15	a) 129-134 b) 112-116	T16	a) 126 b) 109	(7-11) Vs. (12)
85%	85%	**G2**, G4, G8	T17	a) 128-130 b) 134	T18	a) 112-114 b) 116	(7-10) Vs. (11)
93%	93%	G4, **G5**, G6, G7, G8	T19	a) 32 b) 30	T20	a) 27 b) 25	(13) Vs. (14)
93-94%	93-94%	**G4**	T21-T22-T23-T24 a) 290, 281, 280, 283 b) 192, 189, 192, 190				(7-10)

If a particular branching point can be explained through the more than one chemical group transitions, then maximum hitting group is given the higher priority which is listed and highlighted. Say, for the branching point at 76% (Table 12) which can be explained through the hitting groups G2, G5, G7 and G8 where G7 is highlighted as it contains the maximum number of distinct range with respect to others i.e. a) 83 and b) 74 for left sub-tree (T5) and a) 67-72 and b) 64-68 for right sub-tree (T6). Therefore, we find among the non-muscle group, SM MyHc is more hydroxyl containing than other two members (NMHC II-A and NMHC II-B) of this group. So in similar fashion, other branches can also be explained.

### Commonality among NMII family members

We are interested to search new blocks of similar chemical nature which may remain same across the myosin family. We are able to detect only a maximum six (6) length patterns without repetitions in all members of conventional myosin. Table 13 shows the consecutive distinct chemical patterns of various lengths (especially for length 5 aa and length 6 aa) for conventional myosin. The two unique patterns of lengths 6 aa are shown in Table 14. Our algorithms can also predict unique patterns of lower lengths (< 6 aa) without repetitions. We have also examined the higher length patterns, with repetition of chemical groups, by using Algorithm 3, which are common to all the members of conventional myosin as well as distinct for nonmuscle or muscle groups. It is to be noted that if we consider the pattern of higher length (> 6 aa) with repetition of chemical pattern in a particular block, then the pattern having the length less than or equal to six (≤ 6 aa) in that block must be sub pattern of that block. We find the maximum of 20 aa length pattern considering the repetition of the chemical groups as shown in Table 14. Our algorithm reveals the presence of few patterns which are conserved throughout the myosin family or either the non-muscle or the muscle groups (Table 13).

**Table 13:** Conserved chemical patterns in myosin II all members and comparison between Non-Muscle Vs. Muscle Group.

Length (Number Count)	Pattern of Length 5 aa and 6 aa	Existence of Pattern in Non-Muscle Group (Seq. Nos. 1-4)	Existence of Pattern in Muscle Group (Seq. Nos. 5-14)
6 (2)	352471 378124	Yes	Yes
5 (8)	37812 52471 74281 78124 35247 48532 43827 64837	Yes	Yes
6 (11)	524361 361428 286154 731846 184765 476518 651874 847651 628435 438276 827634	Yes	No
5 (49)	16324 24361 36142 41632 12734 24731 27341 37241 47312 73412 24831 38214 82314 84321 45821 18426 61428 42718 48721 72184 28615 34571 54371 31846 48731 73184 84173 47651 14856 61548 85641 86154 51874 18476 76481 65187 76518 52436 28435 35824 43582 27634 46327 63274 62843 87243 38276 82763 84765	Yes	No
6 (3)	813472 635247 748532	No	Yes
5 (9)	13472 24318 43182 81347 63524 24678 67842 82467 74853	No	Yes

Given an input of pattern length (L), all possible combination of pattern of length L using the numerical number 1-8 are generated without repetition using Algorithm 2.

**Table 14:** Two unique patterns of length 6 aa and one pattern of length 20 aa common to myosin 14 members, their position and corresponding amino acids.

Seq. Nos.	Pattern of length 6 aa		Pattern of length 20 aa
	352471	378124	34842742818772342342
	Position-Sequence	Position-Sequence	Position-Sequence
1	560-FPKATD	497-YTNEKL	248-FGNAKTVKNDNSSRFGKFIR
2	541-FPKATD	478-YTNEKL	228-FGNAKTVKNDNSSRFGKFIR
3	534-FPKATD	471-YTNEKL	221-FGNAKTVKNDNSSRFGKFIR
4	541-FPKATD	478-YTNEKL	228-FGNAKTVKNDNSSRFGKFIR
5	552-FPKATD	492-FTNEKL	243-FGNAKTLRNDNSSRFGKFIR
6	543-FPKASD	483-FTNEKL	233-FGNAKTLRNDNSSRFGKFIR
7	543-FPKATD	483-FTNEKL	233-FGNAKTVRNDNSSRFGKFIR
8	543-FPKATD	483-FTNEKL	233-FGNAKTVRNDNSSRFGKFIR
9	543-FPKATD	483-FTNEKL	233-FGNAKTVRNDNSSRFGKFIR
10	543-FPKATD	483-FTNEKL	233-FGNAKTVRNDNSSRFGKFIR
11	541-FPKATD	481-FTNEKL	231-FGNAKTVRNDNSSRFGKFIR
12	542-FPKATD	482-FTNEKL	232-FGNAKTVRNDNSSRFGKFIR
13	540-FPKATD	480-FTNEKL	230-FGNAKTVRNDNSSRFGKFIR
14	541-FPKATD	481-FTNEKL	231-FGNAKTVRNDNSSRFGKFIR

First two patterns of length 6 aa are without repeating of chemical groups (using Algorithm 2) and last pattern of length 20 aa are with the repeating of chemical groups (using Algorithm 3).

The interesting point is to note that the patterns which are conserved throughout the myosin family are mostly located near the ATP binding region. This finding pushes us to hypothesize that these patterns of amino acids must play some important role during ATP binding process. The other different patterns which are specific for any particular group (either non-muscle or muscle) may be responsible for their groups’ specific functions. The entire 5 length patterns (Table 13) with their location and corresponding amino acids sequence of myosin II fourteen members are listed in S2 Table.

Our methods are also able to detect few homologous commonalities in the sequence patterns of myosins, like serine (S) changed to threonine (T). In our conventional sequence matching program, we describe these as different amino acids whereas chemically both are hydroxyl containing and therefore having the same chemical property. We can say that these patterns are chemically conserved through evolutions and may be true that they are doing the same function in different proteins. Head domain of myosin II have 3 broad sub domains: i) ATP Binding Site- It has two sub domains, a) Switch-1 and b) Switch-2 [27], ii) Actin Binding Site and iii) IQ domain. Various unique patterns from Table 13 are shown in Table 15 with their respective positions.

**Table 15:** Specific patterns of the amino acids and their location into different sub-domains of the myosin II head domain.

Seq. Nos.	Length (aa)	Pattern	ATP Domain	Switch-1	Switch-2	Actin Domain
**1-14**	5	74281	Yes	Yes		
	5	48532	Yes			
**1-4**	6	651874, 847651, 476518	Yes			
	5	16324, 41632, 24731, 37241, 47312				
	5	63274				Yes
**5-14**	6	813472, 748532	Yes			
	5	74853, 81347, 13472				
	5	24678, 67824				Yes

### Commonality among MYOI family members

Similar to myosin II, applying Algorithm 2 and Algorithm 3 among the MYOI family members, we are able to detect a block of maximum length 10 aa and three blocks of second maximum of length 9 aa with the repeating of chemical groups and two blocks of maximum length 5 aa without repeating the chemical groups as shown in Table 16. Among the biggest four blocks with the repetition of the chemical groups, the first, second and fourth blocks are located in the ATP binding region and the third block is located in converter sub-domain. And among the last two blocks without repetition the chemical groups, first one is located in Actin binding site (region of interest) and last one is located in purine loop.

**Table 16:** Conserved chemical patterns in MYOI family members, their position and original amino acids sequences.

Seq. Nos.	Pattern of length 10 aa and 9 aa				Patterns of length 6 aa	
		444143431	424224434	344127244	64258	74853
	Pos.-Seq.	Pos.-Seq.	Pos.-Seq.	Pos.-Seq.	Pos.-Seq.	Pos.-Seq.
1	141-VLEAFGNAKT	377-GVLDIYGFE	618-VRVRRAGYA	181-YLLEKSRLV	588-CIKPN	47-SVNPY
2	148-VLEAFGNAKT	384-GVLDIYGFE	625-VRVRRAGYA	188-YLLEKSRVV	595-CIKPN	54-SVNPY
3	144-VLEAFGNAKT	380-GVLDIYGFE	619-VRVRRAGFA	184-YLLEKSRVI	589-CIKPN	48-SVNPY
4	144-VLEAFGNART	392-GVLDIYGFE	631-VRVRRAGFA	184-YLLEKSRVL	601-CIKPN	48-SVNPY
5	180-VLEAFGNAKT	418-GLLDIYGFE	655-LRVRRAGFA	220-YLLEKSRVV	625-CIKPN	86-SVNPY
6	145-VLEAFGNART	388-GLLDIYGFE	625-LRVRRAGFA	185-YLIEKSRVV	595-CIKPN	51-SVNPY
7	152-LLEAFGNAKT	385-GVLDIYGFE	616-IRVRRAGYA	192-FLLEKSRVV	586-CIKPN	58-SVNPF
8	150-LLEAFGNAKT	383-GVLDIYGFE	614-IRVRRAGFA	190-FLLEKSRVV	584-CIKPN	56-SVNPF

### Common motif between myosin II and KIF1A class of Kinesin

To further illustrate and validate our method beyond the same protein group, we compare the protein sequences of the head domain of Kinesin family and myosin II family. Kinesin motor domain is much smaller than the myosin’s (340 vs. 860 amino acids). Although these two proteins share no amino acid identity, as determined by the computational algorithm programs, research on their crystal structure reveals a striking similarity between them. The structural overlap points out some short stretches of sequence conservations [32, 43]. Our algorithm reveals the presence of an octamer with repetition (“84268444”) in both conventional myosin II and the KIF1A class of kinesins. In myosins, this sequence is present in the converter domain, where as in kinesins, it is present in the neck linker region [44]. These sequences and their corresponding positions are shown in Table 17. When sequences of other classes of kinesin are compared with these myosins, this motif is not found suggesting that this common feature is unique to the conventional myosin and KIF1A class of Kinesins.

**Table 17:** Common motifs Myosin II and KIF 1A class of Kinesin, their position and sequences.

Protein	Position-Sequence	Protein	Position-Sequence
**KIF1A**	353-QIRCNAVI	**MYH2**	698-QLRCNGVL
**KIF1B**	347-QIKCNAVI	**MYH4**	696-QLRCNGVL
**KIF1C**	347-QIRCNAII	**MYH8**	695-QLRCNGVL
**MYH14**	715-QLRCNGVL	**MYH1**	696-QLRCNGVL
**MYH11**	698-QLRCNGVL	**MYH3**	693-QLRCNGVL
**MYH9**	691-QLRCNGVL	**MYH13**	696-QLRCNGVL
**MYH10**	698-QLRCNGVL	**MYH7**	692-QLRCNGVL
**MYH15**	704-QLRCNGVL	**MYH6**	694-QLRCNGVL
**MYH7B**	699-QLRCNGVL		

### Common motif between myosin II and MYOI

Here, we compare the protein sequences between two protein families-the head domain of conventional myosin II family and the unconventional myosin MYOI family. We have shown a block/pattern of length 20 aa (Table 14) which have 100% similarity among the myosin II members, but 80–90% similarity among MYOI members (Table 18). There are blocks of length 9 aa which have 100% similarity in MYOI members, but 89–100% similarity among myosin II members (S3 Table).

**Table 18:** Comparison of common motifs from Myosin II with MYOI class of myosin, their position and sequences.

Seq. Nos.	Similarity (%)	Pattern of length 20 aa
		“34842742818772342342”
		Position-Sequence
MYH14		248-FGNAKTVKNDNSSRFGKFIR
1	85	145-FGNAKTIRNNNSSRFGKYMD
2	90	152-FGNAKTVRNDNSSRFGKYMD
3	85	148-FGNAKTNRNDNSSRFGKYMD
4	80	148-FGNARTNRNHNSSRFGKYMD
5	90	184-FGNAKTLRNDNSSRFGKYMD
6	90	149-FGNARTLRNDNSSRFGKYMD
7	85	156-FGNAKTVRNNNSSRFGKYFE
8	85	154-FGNAKTVRNNNSSRFGKYFE

Here, MYH14 (Seq. No. 1, Table 14) is taken as reference sequence from Myosin II family and Seq. Nos. 1-8 are MYOI family members.

We also check the commonality between the MLCK and ROCK protein families, and Na^+^/K^+^-ATPase and Ca^2+^-ATPase families. Although the MLCK and Rho sequences are highly dissimilar, we find three significant blocks, one block of length 7 aa and two blocks of length 6 aa common between them with 100% similarity (S4 Table). They are located in ATP binding site (starting site of activation loop), start of proton acceptor site and ending site of activation loop. Among the Na^+^/K^+^-ATPase family members, four alpha members are highly similar. We are able to detect the maximum of length 78 aa block located in E1-E2_ATPase region (S5 Table). And the maximum of length 8 aa block is detected among the four beta members of Na^+^/K^+^-ATPase family (S5 Table). Similarly, we find three biggest blocks among the Ca^2+^-ATPase family of length 15 aa, 12 aa and 12 aa (S6 Table). All the blocks from Ca^2+^-ATPase family are almost 80–90% similar while comparing with Na^+^/K^+^-ATPase family and vice versa.

## Discussion

We have documented that our algorithm can detect the chemical commonality among proteins. This is surely beyond the capacity of the existing conventional programs. We validate our method by rerunning it in other group of proteins which have same functional role. Results from these experiments suggest that our algorithm can find many common chemical patterns as a block sequence throughout the family. Our method proves its commonality by identifying the common patterns between myosin II and KIF1A which is the first report as per our knowledge. We extend our study between Myosin I and Myosin II, Rho and MLCK, and Na^+^/K^+^-ATPase and Ca^2+^-ATPase.

For the analysis of any newly determined sequences or their evolutionary ancestry, sequence similarity searching is one of the first and foremost informative steps. By the virtue of modern protein sequence databases and searching programs, like BLAST (units 3.4) [45], PSI-BLAST [45], SSEARCH (unit 3.10) [46, 47], HMMER3 [8, 48] and FASTA (unit 3.9) [49], one can produce a very accurate and comprehensive statistical estimates and can predict more than 80% protein sequence samples that share significant sequence similarity. While searching for the sequence similarity is an effective and reliable strategy for identifying sequences, which share a common evolutionary ancestor and the formation of phylogenetic tree, it also possess a significant limitation. When we find some excess sequence similarity, we imply that they are from common ancestry and homologous to each other, which is not entirely true. Homologous sequences do not always share significant entire sequence similarity, but has significant intermediate sequence and structural similarity. Search tools like BLAST, HMMER or FASTA cannot detect this false negatives (i.e.; homologous sequences with non-significant similarity scores), although they can detect and minimize the false positive [8]. Therefore, the emergence of the study on the alignment free analysis is obvious. Using alignment free methods, the biological sequence was supposed to be transformed into an object for which statistical or algebraic theory may be useful as analytical tools. For analyzing the DNA sequences based on the above mentioned basics, there had been significant number of works for the last three decades. These allow to analyze DNA sequences qualitatively and offer a way of viewing, comparing and sorting various genomic sequences. Although, both DNA and protein sequences belong to a symbolic sequence, there are very few methods for analyzing protein sequences qualitatively. This is mainly because of the extension of protein sequences having increased number of possible alternatives for 20 amino acids. These amino acids are the keys to understand the proteins existing in the cell. So, analysis of these amino acid sequences is an important part for the post-genomic study. Recently, several schemes have been proposed to plot amino acid sequences. They categorized the 20 amino acids into different types, including as a word with three to five different letters. Their processes not only describe amino acid sequence, but also determine the similarity/dissimilarity of different protein sequences [14, 47, 50–57]. However, their methods only consider the physical information of the sequences that are conserved in a particular amino acid sequences. Neither their physicochemical properties nor the properties of the adjacent amino acids are considered in those works. Few groups have used the reduced amino acids alphabets to reduce the sample size. They are able to design native-like protein structure from those reduced amino acids alphabets and they hypothesize that their approach might be useful for structurally related proteins weak sequence identity. But one distinguished limitation on their methods is the limited sample of sequences which can only determine a fraction of total sequences. Further, few group works with the converted amino acid sequences of proteins into three letter sequence based on the hydropathy profile of amino acid and algor a conditional probability as a new invariant for the protein sequences [14]. Their method can also show the distribution of amino acids at different positions in the sequences. But, they cannot predict the pattern of conserved chemical nature in the sequences. Therefore, grouping the amino acids based on their chemical properties may offer a better insight into the comparative study of proteins.

All the designed algorithms follow our categorization of various groups based on chemical properties of individual amino acid. Our method gives the clear explanation of the breaking point of every branch of the phylogenetic tree using the number of distinct chemical group transitions. We are also able to place two new myosins (MYH15 and 7B) in the updated phylogenetic tree (Fig 2) by using percent identity matrix. Based on our designed algorithm we can easily find out the conserved domain/common motif with and without repetition of amino acids. By using the results of Algorithm 2, we have provided several conserved patterns that are common to the NMII family. We find another two significant blocks, one block of length 20 aa maximum of 40% similarity in between non-muscle myosin and muscle myosin group located in ATP binding region and another one block of length 17 aa maximum of 94% similarity located nearer to the ATP binding region (S7 Table, by using Algorithm 3 and 4). This may also suggest that the ATP binding region is more chemically conserved compared to Actin binding region. This commonality of chemical patterns may be useful for predicting the structures of proteins, designing the drugs, inhibitors etc. We have shown a longest block of length 20 aa (34842742818772342342, Table 14) among myosin II members in human. The two sub-blocks from the above block “348427”/FGNAKT and “8772342”/NSSRFGK are fixed amino acids sequence among all the members. There is no-chemical group variation of the sequences of those two sub-blocks. These two sub-blocks are previously described in [58] which is located in the ending of HH (helix H). They have worked with 8 representative sequences (among three from myosin class-II, and two from myosin class-I) from different classes of myosin. As per our hypothesis considering the invariant chemical group, we find that 7*th* and 8*th* positions of 20 aa length block are replaced by V/L and K/R respectively where V or L and K or R respectively from same chemical group. Combining these two positions along with others 5 fixed positions and two sub-blocks, we find the block of length of 20 aa which is chemically conserved across the myosin II 14 members in human and that is new compared to the earlier works. The conserved block of length 10 aa (4414348427, Table 16) from MyoI members, last 6 residues are the first sub-block “348427” as described above and also matched with the previous findings [58]. The first position of the 10 aa length block is varying either V or L, and keeping next three positions fixed, combining the 10 aa length chemically conserved block which is also new observation using our algorithm. Two small sub blocks as “414”/LDI and “431”/GFE are located in between HO and HP reported in [58]. This finding is also matched combining with the biggest conserved block of length 9 aa (“444143431”, Table 16) from MyoI members. Similar is the case for the block of length 9 aa (344127244, Table 16) among MyoI members found by our algorithm, a sub-block is previously reported as “4127244”/LLEKSR in [58] located in between HH and HI among various classes of myosin. Further, we have shown another block of length 9 aa (424224434) which is highly chemically conserved among Myo1 family members but not the conserved as well among various classes of myosin except the position 7*th* which is ‘4’/G as observed in previous report located in between HA′ and HB′ [58]. Therefore our findings of various conserved blocks are bigger in length (aa) and could be responsible for group/family specific functions.

**Fig 3:**
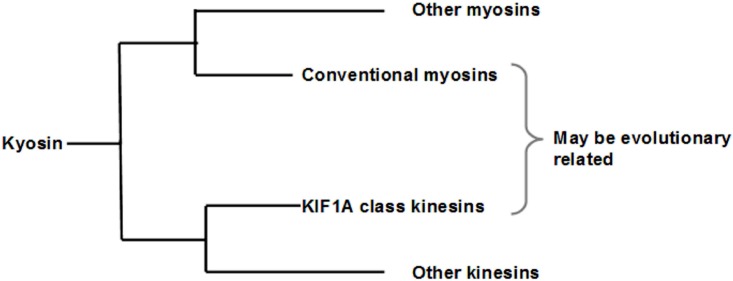
Proposed phylogenetic tree of KIF1A and Conventional myosins.

We have also found that NMII with KIF1A class of Kinesin having a specific pattern which can be studied further for the explanation of biological significance. The neck linker regions of KIF1A class of kinesins are known to be conserved among the family members, but not among the entire super family of kinesins [44]. This would explain the absence of this motif in other kinesins. In kinesin, the neck linker sequence has been shown to be responsible for the generation of mechanical force, in a manner analogous to the converter domains of myosins [59, 60]. This suggests that the KIF1A class of kinesins and conventional myosins may be more closely related than other kinesins and myosins as they share a common motif even though they are from different motor protein families. We hypothesize that this motif must play a functional role in the conversion of chemical energy to mechanical energy. This is probably the reason of the said evolutionarily conserved status (Fig 3).

This result proves the validity of our method in highlighting similarities among proteins with dissimilar amino acid sequences and from different families. The motif shows only 50–62.5% similarity if one goes by amino acid sequence alone, yet the differences in amino acids are minor (leucine and isoleucine, or alanine and glycine) in terms of their chemical natures. Thus the overall chemical nature of the motif is more similar than what alignment of amino acids would imply. It has been already documented that ATP turnover of myosin I follow the same pathway like other myosins [30]. Our current results of having 80–90% similarity in the ATP binding site of myosin I and myosin II also supports that previous finding. Our algorithms also can find the repetition of same pattern in different positions of particular sequence which is quite harder to see by the sequence alignment method. One can easily modify our algorithm based on requirements like changing the number of groups, weight assigning of the groups etc. and this is also applicable even if one needs twenty standard amino acids as twenty groups. Also application can be directed for the pattern analysis among the protein families of various evolutionary species where protein functioning is same. Further, our block finding algorithm (Algorithm 4) can also be used for identifying the highly similar/dissimilar block in the unknown sequences especially for cancer gene or protein family based on neighborhood pattern analysis which is our further investigation. Our confidence relies on this method for assessing the commonalities among various motor proteins and will be quite useful particularly for investigating the function of amino acids during its structural conformation in pathological conditions.

## Supporting Information

S1 Table Chemical group transitions or ordered pairs count of every individual sequence of myosin II fourteen members. (PDF)

S2 Table Various patterns of length 5 aa (from Table 13) and their corresponding amino acid sequences. (PDF)

S3 Table Common blocks/patterns from MYOI family members and their similarity among myosin II family members. (PDF)

S4 Table Common blocks/patterns between MYLK and Rho protein families members. (PDF)

S5 Table Common blocks/patterns among Na^+^-ATPase family members in two groups. (PDF)

S6 Table Common blocks/patterns among Ca^2+^-ATPase family members. (PDF)

S7 Table Two blocks/patterns of length 17 aa and 20 aa and their similarity with non-muscle group and muscle group of myosin II. (PDF)
