# Supplementary material for: Mathematical Characterization of Protein Sequences Using Patterns as Chemical Group Combinations of Amino Acids
Source: PLoS One. 2016 Dec 8;11(12):e0167651. doi: 10.1371/journal.pone.0167651 (PMC5145171; doi:10.1371/journal.pone.0167651)
Supplement: S1 Table — (PDF) [file pone.0167651.s001.pdf]

S1 Table. Chemical group transitions or ordered pairs count of every individual sequence of myosin II fourteen members.

Seq. No-1

	G1	G2	G3	G4	G5	G6	G7	G8
G1	12	16	10	34	5	4	6	15
G2	18	15	11	52	6	5	8	14
G3	6	14	5	24	2	2	13	10
G4	41	44	28	119	16	9	24	30
G5	2	14	3	13	5	2	4	5
G6	3	3	4	13	1	0	4	3
G7	9	11	10	23	7	6	7	4
G8	11	12	5	33	7	2	11	4

Seq. No-2

	G1	G2	G3	G4	G5	G6	G7	G8
G1	17	23	9	29	2	5	6	15
G2	17	15	10	46	4	5	15	17
G3	7	13	6	29	2	3	11	10
G4	39	39	33	99	7	15	26	32
G5	1	7	1	7	3	1	2	5
G6	2	5	5	17	2	1	4	2
G7	14	15	8	28	3	4	9	2
G8	9	12	9	35	5	3	10	5

Seq. No-3

	G1	G2	G3	G4	G5	G6	G7	G8
G1	15	22	11	27	4	5	6	16
G2	17	14	12	50	4	4	13	18
G3	7	16	6	30	2	3	8	9
G4	40	40	29	106	8	14	23	30
G5	2	8	2	9	3	1	1	4
G6	3	5	6	14	2	2	2	4
G7	12	14	7	20	2	5	3	4
G8	10	13	8	34	6	3	11	6

Seq. No-4

	G1	G2	G3	G4	G5	G6	G7	G8
G1	14	25	11	27	4	4	7	15
G2	18	17	12	54	3	5	12	17
G3	7	16	6	29	2	3	10	11
G4	41	40	32	94	7	16	26	29
G5	2	7	1	9	3	1	1	5
G6	5	5	4	16	2	1	2	3
G7	10	15	9	23	2	5	4	4
G8	10	13	9	33	7	2	10	5

Seq. No-5

	G1	G2	G3	G4	G5	G6	G7	G8
G1	15	14	7	40	1	4	9	13
G2	12	19	19	40	6	8	14	9
G3	7	15	9	33	4	7	5	12
G4	46	36	29	99	8	14	35	31
G5	4	8	5	2	2	1	1	2
G6	2	6	6	21	0	1	3	3
G7	10	14	5	38	0	4	7	4
G8	7	15	12	25	5	2	8	6

Seq. No-6

	G1	G2	G3	G4	G5	G6	G7	G8
G1	11	13	8	34	2	5	10	16
G2	10	16	17	48	5	5	15	13
G3	10	13	6	29	2	5	9	14
G4	38	45	30	106	11	11	30	21
G5	4	8	4	8	3	2	2	3
G6	5	5	3	12	0	3	5	4
G7	9	13	9	34	3	3	3	7
G8	12	16	11	21	9	2	7	6

Seq. No-7

	G1	G2	G3	G4	G5	G6	G7	G8
G1	13	19	9	31	4	4	10	12
G2	15	16	14	46	7	6	16	10
G3	7	14	6	29	6	7	8	12
G4	42	41	33	98	5	11	35	25
G5	4	8	6	5	2	2	1	1
G6	2	6	4	13	0	2	3	3
G7	10	13	8	42	1	0	10	9
G8	9	13	9	26	5	0	10	5

Seq. No-8

	G1	G2	G3	G4	G5	G6	G7	G8
G1	13	19	10	28	5	5	11	11
G2	15	17	14	43	8	6	16	10
G3	8	14	6	30	4	7	9	13
G4	40	37	36	92	6	13	35	22
G5	5	8	5	7	2	1	2	1
G6	3	8	4	13	0	3	3	4
G7	10	14	7	44	1	1	8	8
G8	8	12	9	24	6	1	9	7

Seq. No-9

	G1	G2	G3	G4	G5	G6	G7	G8
G1	13	18	9	29	4	5	11	11
G2	15	16	14	45	7	6	16	10
G3	7	14	6	30	5	7	9	13
G4	41	37	35	88	6	13	36	24
G5	5	8	5	6	2	1	2	1
G6	2	8	4	13	0	2	3	4
G7	9	14	9	45	2	0	12	7
G8	8	14	9	24	5	1	9	6

Seq. No-10

	G1	G2	G3	G4	G5	G6	G7	G8
G1	13	19	9	29	4	5	11	11
G2	15	16	14	44	8	6	15	10
G3	7	13	6	30	5	7	8	14
G4	41	38	35	93	6	13	35	22
G5	4	8	5	6	2	2	2	1
G6	2	7	4	14	0	3	4	4
G7	11	14	8	41	1	0	10	8
G8	8	13	9	26	5	1	8	8

Seq. No-11

	G1	G2	G3	G4	G5	G6	G7	G8
G1	14	17	10	30	3	4	14	11
G2	11	18	15	50	6	5	20	9
G3	7	13	5	30	5	8	10	12
G4	42	39	31	87	6	14	34	21
G5	6	7	6	5	2	1	0	2
G6	4	8	4	13	0	1	2	3
G7	10	15	11	39	2	0	11	10
G8	9	17	8	20	6	1	7	7

Seq. No-12

	G1	G2	G3	G4	G5	G6	G7	G8
G1	12	21	10	23	3	6	13	14
G2	14	17	15	43	9	7	10	11
G3	8	15	6	31	5	7	8	11
G4	41	31	31	92	5	17	31	24
G5	5	7	6	7	2	2	1	1
G6	3	7	5	15	1	4	5	5
G7	10	15	8	34	1	0	11	10
G8	9	13	10	27	6	1	10	9

Seq. No-13

	G1	G2	G3	G4	G5	G6	G7	G8
G1	14	19	12	25	3	6	10	13
G2	15	15	13	50	8	5	13	12
G3	9	13	6	32	3	6	8	12
G4	38	43	29	98	7	14	30	26
G5	6	7	5	9	2	1	0	2
G6	3	7	4	16	0	1	3	2
G7	10	16	8	31	3	1	8	5
G8	7	11	12	24	7	1	10	8

Seq. No-14

	G1	G2	G3	G4	G5	G6	G7	G8
G1	15	20	11	25	3	6	10	13
G2	14	16	13	50	7	5	13	13
G3	9	13	7	29	3	6	9	13
G4	39	41	29	94	6	15	32	28
G5	4	7	4	7	2	1	0	2
G6	2	8	4	16	0	1	4	2
G7	13	14	8	35	0	0	7	6
G8	7	12	13	28	7	2	8	8
