# Supplementary material for: Mathematical Characterization of Protein Sequences Using Patterns as Chemical Group Combinations of Amino Acids
Source: PLoS One. 2016 Dec 8;11(12):e0167651. doi: 10.1371/journal.pone.0167651 (PMC5145171; doi:10.1371/journal.pone.0167651)
Supplement: S2 Table — (PDF) [file pone.0167651.s002.pdf]

S2 Table. Various patterns of length 5 aa (from Table 13) and their corresponding amino acid sequences.

Seq. Nos.	Pattern of Length=5							
	**37812**	**52471**	**74281**	**78124**	**35247**	**48532**	**43827**	**64837**
1	497-YTNEK	561-PKATD	253-TVKND	498-TNEKL	560-FPKAT	145-INPYK	505-LFNHT	494-CINYT
2	478-YTNEK	542-PKATD	233-TVKND	479-TNEKL	541-FPKAT	125-VNPYK	486-LFNHT	475-CINYT
3	471-YTNEK	535-PKATD	226-TVKND	472-TNEKL	534-FPKAT	121-INPYK	479-LFNHT	468-CINYT
4	478-YTNEK	542-PKATD	233-TVKND	479-TNEKL	541-FPKAT	125-INPYK	486-LFNHT	475-CINYT
5	492-FTNEK	553-PKATD	248-TLRND	493-TNEKL	552-FPKAT	143-INPYK	620-VFQKS	489-CINFT
6	483-FTNEK	544-PKASD	238-TLRND	484-TNEKL	543-FPKAS	124-INPYK	612-IFQKS	480-CINFT
7	483-FTNEK	544-PKATD	238-TVRND	484-TNEKL	543-FPKAT	126-VNPYK	611-LYQKS	480-CINFT
8	483-FTNEK	544-PKATD	238-TVRND	484-TNEKL	543-FPKAT	126-VNPYK	611-LYQKS	480-CINFT
9	483-FTNEK	544-PKATD	238-TVRND	484-TNEKL	543-FPKAT	128-VNPYK	611-LYQKS	480-CINFT
10	483-FTNEK	544-PKATD	238-TVRND	484-TNEKL	543-FPKAT	126-VNPYK	611-LYQKS	480-CINFT
11	481-FTNEK	542-PKATD	236-TVRND	482-TNEKL	541-FPKAT	126-VNPYK	609-LYQKS	478-CINFT
12	482-FTNEK	543-PKATD	237-TVRND	483-TNEKL	542-FPKAT	126-VNPYK	610-LYQKS	479-CINFT
13	480-FTNEK	541-PKATD	235-TVRND	481-TNEKL	540-FPKAT	125-VNPYK	608-LYQKS	477-CINFT
14	481-FTNEK	542-PKATD	236-TVRND	482-TNEKL	541-FPKAT	125-VNPYK	609-LYQKS	478-CINFT
**Domain**	**After ATP**	**After ATP**	**ATP**	**After ATP**	**After ATP**	**ATP**	**After ATP**	**After ATP**
