# Supplementary material for: Mathematical Characterization of Protein Sequences Using Patterns as Chemical Group Combinations of Amino Acids
Source: PLoS One. 2016 Dec 8;11(12):e0167651. doi: 10.1371/journal.pone.0167651 (PMC5145171; doi:10.1371/journal.pone.0167651)
Supplement: S3 Table — (PDF) [file pone.0167651.s003.pdf]

S3 Table. Common blocks/patterns from MYO1 family members and their similarity among myosin II family members.

Family	Seq. Nos.	Patterns with similarity			
		**Similarity (%)**	**444143431**	**Similarity (%)**	**344127244**
**Myosin II**	1	89	475-GILDIAGFE	100	284-YLLEKSRAI
	2	89	456-GILDIAGFE	100	264-YLLEKSRAI
	3	89	449-GILDIAGFE	100	257-YLLEKSRAI
	4	89	456-GILDIAGFE	100	264-YLLEKSRAV
	5	89	470-GILDITGFE	100	279-YLLEKSRVI
	6	89	461-GVLDIAGFE	100	269-YLLEKSRVI
	7	89	461-GVLDIAGFE	100	269-YLLEKSRVV
	8	89	461-GVLDIAGFE	89	269-YLLEKSRVT
	9	89	461-GVLDIAGFE	89	269-YLLEKSRVT
	10	89	461-GVLDIAGFE	89	269-YLLEKSRVT
	11	89	459-GVLDIAGFE	89	267-YLLEKSRVT
	12	89	460-GVLDIAGFE	89	268-YLLEKSRVT
	13	89	458-GVLDIAGFE	100	266-YLLEKSRVI
	14	89	459-GVLDIAGFE	100	267-YLLEKSRVI
**MYO1**	1	100	377-GVLDIYGFE	100	181-YLLEKSRLV
	2	100	384-GVLDIYGFE	100	188-YLLEKSRVV
	3	100	380-GVLDIYGFE	100	184-YLLEKSRVI
	4	100	392-GVLDIYGFE	100	184-YLLEKSRVL
	5	100	418-GLLDIYGFE	100	220-YLLEKSRVV
	6	100	388-GLLDIYGFE	100	185-YLIEKSRVV
	7	100	385-GVLDIYGFE	100	192-FLLEKSRVV
	8	100	383-GVLDIYGFE	100	190-FLLEKSRVV
**Domain**		**ATP, S-II**		**ATP**	
