# Supplementary material for: Mathematical Characterization of Protein Sequences Using Patterns as Chemical Group Combinations of Amino Acids
Source: PLoS One. 2016 Dec 8;11(12):e0167651. doi: 10.1371/journal.pone.0167651 (PMC5145171; doi:10.1371/journal.pone.0167651)
Supplement: S4 Table — (PDF) [file pone.0167651.s004.pdf]

S4 Table. Common blocks/patterns between MYLK and Rho protein families members.

Family	Seq. nos.	Similarity (%)	Patterns		
			**4244134**	**142518**	**475134**
**MYLK**	1	100	1601-IKLIDFG	1585-DLKPEN	1623-GTPEFV
	2	100	422-VKIIDFG	406-DLKPEN	444-GTPEFL
	3	100	652-IKIIDFG	636-DLKPEN	674-GTPEFL
	4	100	243-IKIIDFG	227-DLKPEN	265-GTPEFL
**Rho**	1	100	212-LKLADFG	198-DVKPDN	236-GTPDYI
	2	100	228-LKLADFG	214-DVKPDN	252-GTPDYI
**Domain**			**ATP, starting site of Activation loop**	**Start of proton acceptor site**	**Ending site of activation loop**
