# Supplementary material for: Mathematical Characterization of Protein Sequences Using Patterns as Chemical Group Combinations of Amino Acids
Source: PLoS One. 2016 Dec 8;11(12):e0167651. doi: 10.1371/journal.pone.0167651 (PMC5145171; doi:10.1371/journal.pone.0167651)
Supplement: S5 Table — (PDF) [file pone.0167651.s005.pdf]

S5 Table. Common blocks /patterns among Na^{+}-ATPase family members in two groups.

Seq. Nos.	Similarity (%)	Pattern
		**34144434444444845144447474647474226422864428414417447774671274747882674426331**
1	100	317-WLEAVIFLIGIIVANVPEGLLATVTVCLTLTAKRMARKNCLVKNLEAVETLGSTSTICSDKTGTLTQNRMTVAHMWFD
2	100	315-WLEAVIFLIGIIVANVPEGLLATVTVCLTLTAKRMARKNCLVKNLEAVETLGSTSTICSDKTGTLTQNRMTVAHMWFD
3	100	307-WLEAVIFLIGIIVANVPEGLLATVTVCLTLTAKRMARKNCLVKNLEAVETLGSTSTICSDKTGTLTQNRMTVAHMWFD
4	100	325-WLEAIIFLIGIIVANVPEGLLATVTVCLTLTAKRMARKNCLVKNLEAVETLGSTSTICSDKTGTLTQNRMTVAHMWFD
**Seq. Nos.**	**Similarity (%)**	**3353342**
5	100	242-YYPYYGK
6	100	227-YFPYYGK
7	100	214-YFPYYGK
8	100	295-YYPYYGK
**Domain**		**E1-E2_ATPase**
