# Supplementary material for: Mathematical Characterization of Protein Sequences Using Patterns as Chemical Group Combinations of Amino Acids
Source: PLoS One. 2016 Dec 8;11(12):e0167651. doi: 10.1371/journal.pone.0167651 (PMC5145171; doi:10.1371/journal.pone.0167651)
Supplement: S6 Table — (PDF) [file pone.0167651.s006.pdf]

S6 Table. Common blocks/patterns among Ca^{2+} -ATPase family members

		Patterns		
	**Similarity (%)**	**444444514454447**	**744671274747**	**814544224144**
1	100	302-LAVAAIPEGLPAVIT	346-SVICSDKTGTLT	706-NDAPALKKAEIG
2	100	302-LAVAAIPEGLPAVIT	346-SVICSDKTGTLT	705-NDAPALKKAEIG
3	100	302-LAVAAIPEGLPAVIT	346-SVICSDKTGTLT	706-NDAPALKKAEIG
4	100	426-VLVVAVPEGLPLAVT	470-TAICSDKTGTLT	800-NDGPALKKADVG
5	100	450-VLVVAVPEGLPLAVT	494-TAICSDKTGTLT	823-NDGPALKKADVG
6	100	424-VLVVAVPEGLPLAVT	468-TAICSDKTGTLT	797-NDGPALKKADVG
7	100	416-VLVVAVPEGLPLAVT	460-TAICSDKTGTLT	788-NDGPALKKADVG
	**Domain**	**Transmembrane**	**Before E1_E2_ATPase**	**-**
