# Supplementary material for: Mathematical Characterization of Protein Sequences Using Patterns as Chemical Group Combinations of Amino Acids
Source: PLoS One. 2016 Dec 8;11(12):e0167651. doi: 10.1371/journal.pone.0167651 (PMC5145171; doi:10.1371/journal.pone.0167651)
Supplement: S7 Table — (PDF) [file pone.0167651.s007.pdf]

S7 Table. Two blocks/patterns of length 17 aa and 20 aa and their similarity with non-muscle group and muscle group of myosin II.

Seq. nos.	Similarity (%)	Pattern 22128718476518744824	Similarity (%)	Pattern 18464837812488338
1	100	381-KRERNTDQATMPDNTAAQKL	94	491-EQLCINYTNEKLQQLFN
2	100	362-KKERNTDQASMPDNTAAQKV	94	472-EQLCINYTNEKLQQLFN
3	100	355-KKERNTDQASMPDNTAAQKV	94	465-EQLCINYTNEKLQQLFN
4	100	362-KKERNTDQASMPENTVAQKL	94	472-EQLCINYTNEKLQQLFN
5	40	201-GKTVNSKHIIQYFATIAAMI	100	486-EQLCINFTNEKLQQFFN
6	40	597-WLEKNKDPLNETVVPIFQKS	100	477-EQLCINFTNEKLQQFFN
7	40	184-GKTVNTKRVIQYFATIAVTG	100	477-EQLCINFTNEKLQQFFN
8	40	184-GKTVNTKRVIQYFATIAVTG	100	477-EQLCINFTNEKLQQFFN
9	40	186-GKTVNTKRVIQYFATIAVTG	100	477-EQLCINFTNEKLQQFFN
10	40	184-GKTVNTKRVIQYFATIAVTG	100	477-EQLCINFTNEKLQQFFN
11	40	184-GKTVNTKRVIQYFATIAATG	100	475-EQLCINFTNEKLQQFFN
12	40	184-GKTVNTKRVIQYFATIAVTG	100	476-EQLCINFTNEKLQQFFN
13	40	206-KKDQSPGKGTLEDQIIQANP	100	474-EQLCINFTNEKLQQFFN
14	40	183-GKTVNTKRVIQYFASIAAIG	100	475-EQLCINFTNEKLQQFFN
**Domain**	**ATP binding site**		**Nearer to the ending of ATP binding site**	
